# Protein tyrosine kinase 2b inhibition reverts niche-associated resistance to tyrosine kinase inhibitors in AML

**DOI:** 10.1038/s41375-022-01687-x

**Published:** 2022-09-02

**Authors:** Catana Allert, Alexander Waclawiczek, Sarah Miriam Naomi Zimmermann, Stefanie Göllner, Daniel Heid, Maike Janssen, Simon Renders, Christian Rohde, Marcus Bauer, Margarita Bruckmann, Rafael Zinz, Cornelius Pauli, Birgit Besenbeck, Claudia Wickenhauser, Andreas Trumpp, Jeroen Krijgsveld, Carsten Müller-Tidow, Maximilian Felix Blank

**Affiliations:** 1 Department of Medicine V, Hematology, Oncology and Rheumatology, University Hospital Heidelberg, Heidelberg, Germany; 2 University of Heidelberg Medical Faculty, Heidelberg, Germany; 3 Division Stem Cells and Cancer, German Cancer Research Center (DKFZ), Heidelberg, Germany; 4 Division Proteomics of Stem Cells and Cancer, German Cancer Research Center (DKFZ), Heidelberg, Germany; 5 Molecular Medicine Partnership Unit (MMPU), University of Heidelberg and European Molecular Biology Laboratory (EMBL), Heidelberg, Germany; 6 European Molecular Biology Laboratory EMBL, Heidelberg, Germany; 7 Department of Pathology, University Hospital Halle (Saale), Halle, Germany; 8 Division of Mechanisms Regulating Gene Expression, German Cancer Research Center (DKFZ), Heidelberg, Germany

**Keywords:** Acute myeloid leukaemia, Preclinical research

## Abstract

FLT3 tyrosine kinase inhibitor (TKI) therapy evolved into a standard therapy in FLT3-mutated AML. TKI resistance, however, develops frequently with poor outcomes. We analyzed acquired TKI resistance in AML cell lines by multilayered proteome analyses. Leupaxin (LPXN), a regulator of cell migration and adhesion, was induced during early resistance development, alongside the tyrosine kinase PTK2B which phosphorylated LPXN. Resistant cells differed in cell adhesion and migration, indicating altered niche interactions. PTK2B and LPXN were highly expressed in leukemic stem cells in FLT3-ITD patients. PTK2B/FAK inhibition abrogated resistance-associated phenotypes, such as enhanced cell migration. Altered pathways in resistant cells, assessed by nascent proteomics, were largely reverted upon PTK2B/FAK inhibition. PTK2B/FAK inhibitors PF-431396 and defactinib synergized with different TKIs or daunorubicin in FLT3-mutated AML. Midostaurin-resistant and AML cells co-cultured with mesenchymal stroma cells responded particularly well to PTK2B/FAK inhibitor addition. Xenograft mouse models showed significant longer time to leukemia symptom-related endpoint upon gilteritinib/defactinib combination treatment in comparison to treatment with either drug alone. Our data suggest that the leupaxin-PTK2B axis plays an important role in acquired TKI resistance in AML. PTK2B/FAK inhibitors act synergistically with currently used therapeutics and may overcome emerging TKI resistance in FLT3-mutated AML at an early timepoint.

## Introduction

Acute myeloid leukemia is the most common acute leukemia in adults and is associated with recurrent mutations. Mutations in the Fms-like tyrosine kinase 3 (FLT3) are the most frequent genetic abnormalities in AML, accounting for approximately 30% of cases, most commonly occurring as FLT3 internal tandem duplication (FLT3-ITD). FLT3 mutations are associated with a more aggressive course of disease and significantly reduced overall survival [1]. Although approved FLT3-inhibitors like midostaurin or gilteritinib improved outcome [2, 3], therapy resistance and relapse remain a central problem in treatment of FLT3-mutated AML. The assumed reason is a small population of cells that escapes chemotherapy by different resistance mechanisms and thus can fuel relapse, often referred to as leukemic stem cells (LSCs). Hence, a better understanding of the mechanisms underlying FLT3-inhibitor resistance and therapeutic approaches to overcome this resistance are urgently needed.

One well-established concept of chemotherapy-resistance in leukemia is cell adhesion-mediated drug resistance (CAM-DR). Early on, it has been shown that AML cells are less prone to drug-induced apoptosis upon co-cultivation with a human bone marrow stromal cell line (HS-5) [4]. Besides their direct involvement in mediating intercellular binding or the interaction with the extracellular matrix, cell adhesion molecules (CAMs) as single-pass transmembrane molecules play a central role in translating external stimuli into intracellular signaling cascades [5]. CAMs are widely expressed on leukemia cells and cells of the bone marrow niche and have been shown to play an important role in LSC homeostasis, their protection, and their escape from standard chemotherapy [6, 7].

The focal adhesion protein leupaxin (LPXN) is a transcriptional coactivator and has been shown to promote cell migration, adhesion and invasion in different entities, such as prostate or breast cancer [8, 9].

Proline-rich tyrosine kinase 2 (PTK2B/PYK2) is a focal adhesion tyrosine kinase that shares structural and sequence homology with focal adhesion kinase (FAK) and has also been shown to promote cell migration and invasion in a variety of cancers, such as breast cancer [10].

In this study, we assessed global differences in protein homeostasis upon emerging and established midostaurin-resistance in FLT3-mutated AML cells. We found that LPXN was phosphorylated by the tyrosine kinase PTK2B and that both were induced early upon acquired midostaurin-resistance. In line with the importance of CAMs in drug-resistance and the interplay of leukemia cells with the bone marrow niche, resistant cells showed significantly altered migration and adhesion properties, and both LPXN and PTK2B were upregulated in LSCs of FLT3-mutated patients. We showed that PTK2B/FAK inhibition by PF-431396 or defactinib synergized with TKIs and other commonly used chemotherapeutic agents in FLT3-mutated AML. The addition of a PTK2B/FAK inhibitor suppressed midostaurin-resistance and reverted resistance-associated phenotypes and pathways. Co-culture assays of an FLT3-ITD AML cell line with mesenchymal stroma cells further linked this novel drug combination to altered niche interactions. Treatment of AML xenografts in NSG mice confirmed the synergy between FLT3- and PTK2B/FAK-inhibition in-vivo.

## Materials and Methods

### Cell culture, primary AML samples, and treatments

MV4-11, HL-60, MOLM-13, OCI-AML2, OCI-AML3, and Kasumi-1 human AML cell lines, HEK293T and HS-5 mesenchymal stroma cell lines were purchased from DSMZ and cultured in MEM Alpha (OCI-AML3), IMDM (MV4-11, HEK293T), DMEM (HS-5) or RPMI1640 (other cell lines) medium (21875091, Thermo Fisher Scientific, Waltham, Massachusetts, USA) supplemented with 10% (HL-60, MV4-11, HEK293T, HS-5) or 20% (other cell lines) FBS (Bio&SELL GmbH, Feucht, Germany). MV4-11 cells tested negative for mycoplasma contamination. Resistant cell lines were generated by treating cells twice per week with increasing doses of PKC-412/midostaurin (M1323-1MG, Sigma Aldrich). The PTK2B inhibitors PF-431396 and defactinib (VS-6063) were obtained from Sigma Aldrich (PZ0185) and from Biozol, Eching, Germany.

Primary AML samples were obtained from bone marrow or peripheral blood of patients with informed consent. The ethics committee of Heidelberg University approved the study (S-686/2018). Mononuclear cells were isolated by Ficoll density gradient centrifugation.

### Multilayered proteome analyses

See Supplementary methods

### Viability assays

Cell viability was assessed after seeding cells into 96 well plates with a density of 1 × 10^4^–5 × 10^4^ cells per well for cell lines and patient samples. After 72 h of treatment cells were stained with trypan blue (T8154, Sigma Aldrich), and viable cells were counted. Alternatively, cells were stained with MTS reagent (G3582, Promega) and analyzed on a Tecan plate reader.

### Co-immunoprecipitation

Proteins were crosslinked with 3 mM DSS (21555, ThermoFisher Scientific) for 30 min at room temperature. Immunoprecipitation of LPXN was performed using Dynabeads® Protein G Immunoprecipitation Kit (Life Technologies, Darmstadt, Germany) according to manufacturer’s instructions and using the indicated antibodies. Precipitated proteins were eluted in urea buffer 6 M for mass spectrometry analysis or SDS sample buffer for western blot detection.

### Immunoblotting

Cells were pelleted and lysed and equal protein amounts were subjected to SDS-PAGE and then transferred onto a nitrocellulose membrane. The following primary antibodies were used: anti-LPXN (ab181621, Abcam; LS‑C313296, LSBio), anti-ß-actin (A5441, Sigma Aldrich), anti-PYK2 (ab81266, Abcam), anti-GAPDH (2118S, CST), anti-phosphotyrosine (ab10321, Abcam), anti-V5 tag (ab9116, Abcam), anti-FAK antibody (3285, CST).

### Forced LPXN and PTK2B expression

Lentiviral vectors encoding for LPXN or PTK2B were generated by PCR amplification from cDNA and cloned into pCDH-CMV-MCS-EF1-GFP, pCDH-CMV-MCS-EF1-RFP vector (CD511B-1, CD512B-1, Biocat). HEK293T cells were transfected with lipofectamine 3000 (L3000001, Invitrogen) according to manufacturer’s protocol.

### Generation of single and simultaneous knockout of FAK and PTK2B

SgRNA target sequences (FAK: GAATCAGTTACCTAACGGACA), (PTK2B: GA TGA GGG TAT AAA GGA CCG G) were cloned into the pL-CRISPR.EFS.GFP vector (57818, Addgene). AML cell lines were transduced by lentiviral transduction.

### RNA extraction, reverse transcription, and quantitative PCR

RNA was isolated with Direct-zol RNA MiniPrep (ZYM-R2050, Biozol) and transcribed into cDNA with random hexamers (N8080127, Life Technologies). Real-time PCR was performed using the following primer pairs: LPXN (fwd: GGAAGGTGATCCATGCTCTAGG; rev: AAGAAGGGACTGGAGCCAATC), PTK2B (fwd: CGGTACATTGAGGACGAGGA; rev: TTCTCCAGCCAGAAGAAGGG), GAPDH (fwd: CATCACTGCCACCCAGAAGAC; rev: CAGTGAGCTTCCCGTTCAGC).

### Colony formation assays

For cell line experiments, 300 cells were seeded into 500 µl methylcellulose (04230, STEMCELL technologies) supplemented with penicillin/streptomycin and the indicated drugs. After seven to eight days colonies were counted.

### Cell adhesion assays

Cell adhesion assays were performed using CytoSelect™ 48-well cell adhesion assay (CBA-050-CB, Biocat) according to the manufacturer´s protocol. MV4-11 cells were treated with PKC-412 and PF-431396 for 24 h before starting the assay. 250.000 cells per well and replicate were used.

### Cell migration assays

Migration assays were performed with CytoSelect™ 24-well Cell Migration (CBA-100, Biocat) kits. 500.000 cells were used per replicate. Cells were allowed to migrate for 24 h. Readout was performed by light microscopy and cell extraction according to manufacturer´s protocol.

### Co-culture assays

See Supplementary methods

### In-vivo experiments

NOD.*Prkdc*^*scid*^.*Il2rg*^*null*^ (NSG) mice were bred and housed under specific pathogen-free conditions at the central animal facility of the German Cancer Research Center (DKFZ). Animal experiments were conducted in compliance with ethical regulations and experiments were approved by the Regierungspräsidium Karlsruhe (G-140-21).

Female mice 8-12 weeks of age were sublethally irradiated (175 cGy) 24 h before injection of 1 × 10^6^ MV4-11R cells (transduced with pCDH-EF1-Luc2-P2A-tdTomato, plasmid #72486, Addgene) into the tail vein. 5 mice per treatment group were chosen to make statistically reliable conclusions. Only mice with no engraftment at day 7 were excluded. Investigators were not blinded. Leukemic engraftment was evaluated by bioluminescence imaging. Treatment was performed via daily oral gavage with 8 mg/kgBW of gilteritinib, 50 mg/kgBW of defactinib, combination therapy or vehicle. Treatment was initiated 7 days post-injection after leukemic engraftment was confirmed by bioluminescence imaging. The endpoint was reached when mice started showing leukemia related symptoms and they were sacrificed. Bioluminescence images were analyzed by annotating the mice with MITK workbench v2022.04 (DKFZ) and photons per second were calculated.

### Graphical outlines

Workflow schemes were created with https://BioRender.com.

### Statistical analysis

All statistical tests were performed using R version 4.5 and R-studio version 1.13 or GraphPad Prism version 9.3.1.

Values are presented as mean ± s.d. of replicates. Two-tailed Student’s test was used to determine statistical significance unless stated otherwise. Nonlinear-regression analysis was performed for calculation of IC_50_ values. *p*-values < 0.05 were considered to be statistically significant. Synergy scores were computed according to the Bliss Independence Model and the Zero Interaction Potency Model (ZIP) [11] with the synergyfinder R-package version 2.4.13 [12]. Log-rank (Mantel-Cox) test was used to evaluate Kaplan–Meier time to leukemia-symptom related endpoint analysis.

## Results

### LPXN is induced during acquired midostaurin-resistance

**Fig. 1:**
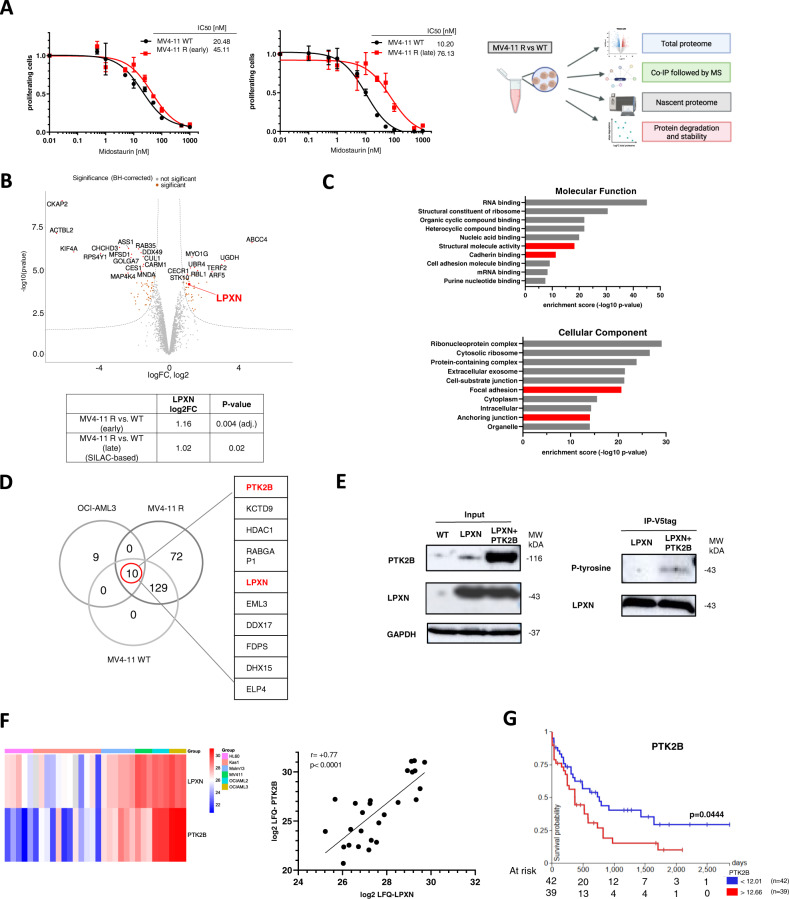
LPXN is induced during acquired midostaurin-resistance and is phosphorylated by PTK2B. **A** MV4-11 cells were exposed to increasing concentrations of midostaurin for several weeks. Acquired midostaurin-resistance was analyzed by MTS assays. MV4-11 cells with approximately doubled IC_50_ were termed MV4-11R early (left), MV4-11 cells with approximately 7-fold higher IC_50_ are referred to as MV4-11R late (middle). Depicted are means from technical triplicates ± SD. Proteomics workflow scheme of MV4-11R vs WT cell analyses (right). **B** Total proteome analysis by mass spectrometry of MV4-11 parental and MV4-11R (early). Volcano plot of differentially expressed proteins in MV4-11R versus parental cells, statistically significant proteins are labelled in orange (log2FC >0.65, adj. p-value< 0.05). LPXN is highlighted in red. **C** Co-immunoprecipitation of endogenous LPXN in MV4-11R cells. GO pathway analysis of molecular function (upper) and cellular component (lower) of proteins interacting with LPXN in MV4-11R cells. **D** Co-immunoprecipitation of endogenous LPXN using two different LPXN antibodies (Abcam, LSBio) in MV4-11 WT, R, and OCI-AML3 cells. Co-purified proteins were analyzed by mass spectrometry. IP with IgG serving as control. Significantly enriched proteins/overlap in all three cell lines are shown. **E** HEK293T cells were transfected with plasmids encoding V5-LPXN, PTK2B, or empty vector. After immunoprecipitation of LPXN with a V5-tag antibody, tyrosine phosphorylation of LPXN was assessed by western blotting in the presence or absence of PTK2B co-expression. V5-LPXN and empty vector-transfected cells served as control. **F** Total proteome analyses in different AML cell lines: FLT3-mutated (MOLM-13, MV4-11) and FLT3 wildtype (Kasumi-1, HL-60, OCI-AML2, OCI-AML3). Left: Heatmap of LPXN and PTK2B expression across all cell lines. Right: Correlation of LPXN and PTK2B expression in the proteomic datasets of the different AML cell lines (Pearson r = 0.7680, p < 0.0001). **G** Kaplan–Meier plot for overall survival of patients with low and high PTK2B mRNA expression (TCGA LAML data set, UCSC Xena browser). Statistical significance was determined by log-rank test.

We exposed MV4-11 cells to increasing concentrations of midostaurin and analyzed acquired drug-resistance by MTS assays. We performed total proteome analysis by mass spectrometry in parental and resistant cells at the timepoint of approximately doubled IC_50_ for midostaurin (20 nM vs. 45 nM, early resistance, upon three months of drug treatment) and 7-fold increase in IC_50_ (10 nM vs. 76 nM, late resistance, upon five months of drug treatment, Fig. 1A).

Numerous proteins showed altered levels in early resistant cells compared to wildtype cells (150 downregulated, 104 upregulated (log2FC >0.65), adj. *p* value < 0.05 Fig. 1B). Many proteins involved in cell cycle regulation were profoundly downregulated, whereas factors implicated in the cellular immune response were upregulated. Despite not reaching significance threshold, also pathways with well-established roles in drug-resistance showed higher expression levels in resistant cells, such as mTOR signaling (Supplementary Fig. S1A) [13]. Gene ontology (GO) analysis revealed that among upregulated proteins, factors located in the plasma membrane were significantly enriched (Supplementary Fig. S1A). Given the known concept of CAM-DR we assessed whether factors with implications in intercellular contact or signaling were dysregulated. We identified leupaxin (LPXN), a transcriptional coactivator and regulator of cell migration and adhesion, to be induced in both early- and late-resistant MV4-11 cells (Fig. 1B).

### LPXN interacts with and is phosphorylated by PTK2B

We performed co-immunoprecipitation (co-IP) experiments of LPXN in parental and midostaurin-resistant AML cells, as well as in FLT3-wildtype cells with high LPXN expression (OCI-AML3) and analyzed LPXN-interacting proteins by mass spectrometry. We used two different antibodies targeting endogenous LPXN with similar pulldown efficiencies to exclude antibody-related effects.

Interestingly, focal adhesion and cell adhesion molecule binding ranked among the top GO terms of LPXN-interacting proteins (Fig. 1C). Other co-purified proteins, such as fermitin family homolog 3 (FERMT3) and talin-1 (TLN1), have also been described to be involved in the connection of cytoskeletal structures to the plasma membrane and cell adhesion (Supplementary Fig. 1B) [14–16]. Notably, we previously identified leupaxin within a resistance-associated EZH2 complex in AML [17]. The co-IP data analysis resulted in the identification of overall 211 direct and indirect interaction partners of LPXN, with ten proteins significantly enriched in all three co-immunoprecipitation experiments with both LPXN antibodies.

One of those ten high-confidence interaction partners of LPXN was the tyrosine kinase PTK2B (Fig. 1D). These findings are in line with a previous study showing LPXN-PTK2B interaction in leukocytes [18]. Comparison of LFQ (label-free quantification)-based intensities suggested that the interaction of LPXN with PTK2B was enhanced in resistant MV4-11 cells (Supplementary Fig. S1C). Given that LPXN is a reported phosphoprotein we addressed the question whether LPXN is a substrate of PTK2B. Only upon co-expression of PTK2B a phosphotyrosine signal corresponding to phospho-LPXN was detected by western blot after immunoprecipitation of ectopically expressed LPXN (Fig. 1E). Accordingly, LPXN is a novel substrate of PTK2B.

### Expression levels of LPXN and PTK2B closely correlate in AML

Next, we characterized LPXN and PTK2B expression by total proteome analyses in two FLT3-mutated (MV4-11, MOLM-13) and four FLT3-wildtype (Kasumi-1, HL60, OCI-AML2, and OCI-AML3) AML cell lines. We observed a close correlation between LPXN and PTK2B expression with particularly high levels in FLT3-mutated cell lines, as well as OCI-AML2 and 3 (Fig. 1F). This correlation was also observed, due to the concomitant upregulation of both proteins, in midostaurin-resistant MV4-11 cells. Interestingly, in previously published total proteomics data comparing LSCs and non-LSCs, we found that both PTK2B and LPXN were significantly overexpressed in LSCs of FLT3-mutated but not FLT3 wildtype patients (Supplementary Fig. S1D) [19]. Next, we examined LPXN and PTK2B expression in tissue micro arrays (TMA) of bone marrow biopsies of 190 AML patients at diagnosis (Supplementary Fig. S1E). LPXN was detected in 88 samples mostly with very low expression levels. PTK2B showed an all-or-none pattern of expression. Despite the low expression profiles, the data confirmed the correlation between LPXN and PTK2B in primary samples (Pearson r = +0.39, *p* < 0.0001) (Supplementary Fig. S1E–H). In the TCGA-AML dataset high PTK2B expression was associated with worse overall survival (Fig. 1G, *p* = 0.044). These findings were in line with a suspected function in therapy resistance and potentially LSC homeostasis. A similar trend was observed for LPXN (Supplementary Fig. S1I, *p* = 0.157).

### Resistance-associated alterations in protein homeostasis are reverted by PTK2B inhibition

**Fig. 2:**
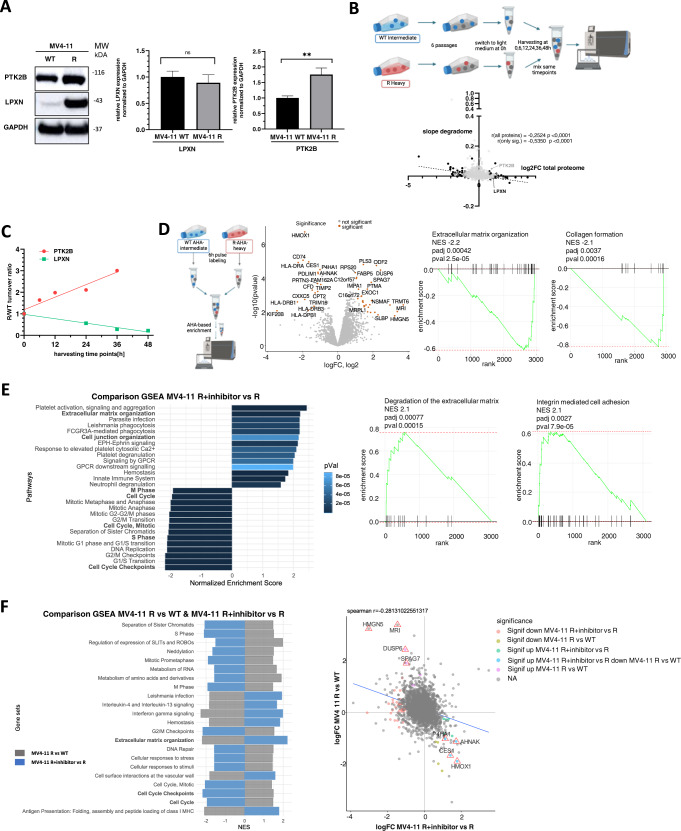
Alterations in protein homeostasis upon midostaurin-resistance and PTK2B inhibition. **A** Left: LPXN and PTK2B upregulation in MV4-11R (early) cells was confirmed by western blot. Data are representative for two independent experiments. Right: mRNA levels of LPXN and PTK2B in MV4-11 parental and MV4-11R (early) cells as analyzed by RT-qPCR. Depicted are means from triplicates ± SD. Statistical significance was assessed using unpaired two-tailed students *t*-test. **p = 0.0041. **B** Analyses of protein stability in leukemia cells. Outline (upper): MV4-11 parental and MV4-11R cells were labeled with SILAC medium (intermediate (IM) and heavy (HV)) for six passages. After switching back to light-medium, cells were harvested at different time points and HV/IM ratios were assessed by LC-MS/MS analysis over time to assess for differences in protein stability/degradation. Lower: Correlation of degradome with total proteome of MV4-11R (late) cells. Degradation slope was calculated as turnover ratio over the different harvesting time points and compared to total proteome (all 2043 proteins are labeled in grey, significant proteins (164) were labeled in black). Pearson correlation coefficient r and p-values are depicted. **C** Resistant/parental turnover ratios for LPXN and PTK2B at different harvesting time points, normalized to 1 at 0 h. **D** Nascent proteomics of MV4-11 parental and resistant cells. Left: schematic depiction of AHA-SILAC pulse labeling and enrichment of newly synthesized proteins. Middle: volcano plot of differentially translated proteins, significantly altered proteins are labelled in orange (log2FC >1, *p*-value < 0.05). Right: GSEA for proteins involved in extracellular matrix organization and collagen formation in MV4-11R vs WT cells. **E** Nascent proteomics of MV4-11R cells with or without PF-431396 treatment (16 h, 300 nM). Left: GSEA of changes in translation upon PTK2B inhibition. Right: GSEA for proteins involved in degradation of extracellular matrix organization and integrin-mediated cell adhesion in MV4-11R treated with 300 nM PF-431396 compared to MV4-11R cells with mock treatment (R). **F** Left: Comparison of up- and downregulated gene sets of nascent proteomics experiment of MV4-11R vs WT (grey) and MV4-11R+ inhibitor vs R (blue). PTK2B inhibitor treatment reverts major effects of R vs. WT on nascent proteome level. Right: Spearman correlation of nascent proteome of MV4-11R+ inhibitor vs R with MV4-11R vs WT.

Proteomic analyses revealed an upregulation of both LPXN and PTK2B upon midostaurin-resistance. Whereas PTK2B was upregulated on the transcriptional level, LPXN mRNA levels in parental and resistant MV4-11 cells were unchanged, indicating post-transcriptional regulation (Fig. 2A). In order to test whether LPXN is stabilized by the interaction with, or phosphorylation by, PTK2B we used a SILAC-based approach to assess for differences in protein degradation and/or stability in MV4-11 parental and resistant cells (Fig. 2B). Overall, proteins with higher expression levels in resistant cells rather had a shortened half-life (negative degradation slope) and vice versa (Fig. 2B). LPXN turnover was increased in resistant cells, suggesting that enhanced translation and not stabilization or decreased degradation were responsible for LPXN upregulation in early midostaurin-resistance. PTK2B stability, on the other hand, was increased in resistant cells, indicating that it was upregulated both on the transcriptional and post-translational level (Fig. 2C). Further, we performed nascent proteomics experiments in parental and resistant MV4-11 cells. Consistent with the steady-state proteome data, proteins involved in ribosomal function and translation, exhibited altered synthesis rates, as well as proteins which are important for degradation or organization of the extracellular matrix (Fig. 2D, Supplementary Fig. S2A). LPXN showed higher synthesis rates in resistant cells as compared to parental cells (Supplementary Table S1). Overall, these data provide evidence that the increase of LPXN expression depended on enhanced translation.

Next, we analyzed the impact of PTK2B inhibition on midostaurin-resistant cells. For this, we performed nascent proteomics on resistant MV4-11 cells which were exposed to the combined PTK2B/FAK inhibitor PF-431396 for 16 h. The treatment affected the translation rate of numerous proteins, including factors involved in cell-cell communication, cell junction organization and various signaling cascades (Fig. 2E, Supplementary Fig. S2B). Remarkably, proteins involved in cell cycle regulation and extracellular matrix organization were most prominently affected by PF-431396 treatment. These protein groups were also significantly affected in our differential expression analysis between parental and resistant cells. In the majority of cases PF-431396 treatment resulted in complete compensation of the effects on protein translation observed in resistant compared to parental cells (Fig. 2F, Supplementary Fig. S2C). Exemplarily, resistant cells showed higher synthesis of HMGN5, MRI, DUSP6, or SPAG7, while PF-431396 exposure resulted in suppression of their translation (Fig. 2F). These significant and opposing overlaps between differential protein expression in resistant versus parental cells and the effects of PTK2B inhibition on protein synthesis suggest that PTK2B upregulation constitutes an important step in the emergence of drug-resistance.

### PTK2B/FAK inhibitor PF-431396 abolishes enhanced cell migration in midostaurin-resistant cells

**Fig. 3:**
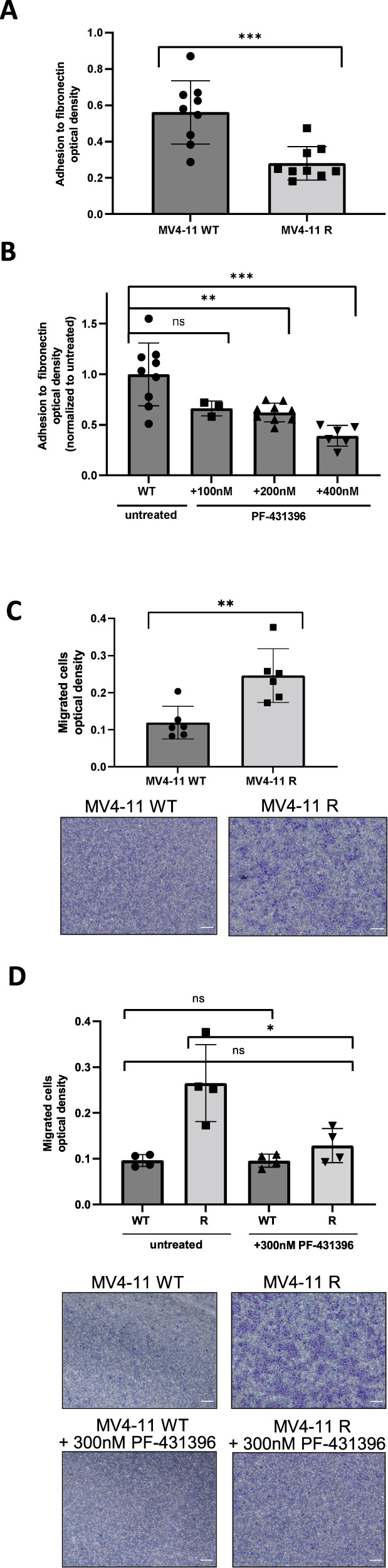
PTK2B/FAK inhibitor PF-431396 abolishes enhanced cell migration in midostaurin-resistant cells. **A** Adhesion to ECM proteins in MV4-11 parental and resistant cells was assessed by fibronectin adhesion assays. Depicted are means from triplicates of three biological replicates ± SD. Statistical significance was assessed using unpaired two-tailed students t-test. ***p = 0.0006. BSA-coated wells served as control. **B** Adhesion assays were performed with MV4-11 parental cells which were treated with different concentrations of PF-431396 for 24 h prior to the assay at equal density. Untreated cells serving as control. Depicted are means from triplicates of at least two biological replicates ± SD. Statistical significance was assessed using unpaired two-tailed students *t*-test. ***p = 0.0005, **p = 0.003, ns = not significant. **C** Cell migration assays were performed with MV4-11 parental and MV4-11R cells which migrated for 24 h. After staining and washing, migratory cells were measured by cell counting (Supplementary Fig. S3A) and after extraction by colorimetry/optical density (upper). Depicted are means from duplicates of three biological replicates ± SD. Pictures were taken after staining of migratory cells (lower). Scale bars represent a length of 0.12 mm. Statistical significance was assessed using unpaired two-tailed students *t*-test. **p = 0.0043. **D** Cell migration assays were performed as in 3C with MV4-11 parental and resistant cells which were treated with 300 nM PF-431396. After staining and washing, migratory cells were measured by cell counting (Supplementary Fig. S3B) and colorimetry/optical density (upper). Depicted are means from duplicates of two biological replicates ± SD. Pictures were taken after staining of migratory cells (lower). Scale bars represent a length of 0.12 mm. Statistical significance was assessed using unpaired two-tailed students *t*-test. *p = 0.0250, ns not significant.

We investigated whether the upregulation of LPXN and PTK2B in resistant cells was accompanied by alterations in cell adhesion and migration. MV4-11R cells lost adhesive strength to fibronectin-coated plates compared to parental cells (Fig. 3A). Treatment of cells with the PTK2B/FAK inhibitor PF-431396 decreased cell adhesion in a dose-dependent manner (Fig. 3B). In transwell migration assays, on the other hand, we found midostaurin-resistant cells to exhibit profoundly increased migration (Fig. 3C, Supplementary Fig. S3A). Alterations in cell adhesion or migration suggested functions in niche interactions, which are closely linked to LSC biology [20, 21]. As PTK2B and LPXN are highly expressed in LSCs of FLT3-mutated patients, we wanted to assess whether addition of a PTK2B inhibitor reduced this resistance-associated increase in cell migration. Notably, 300 nM of PF-431396 alone reverted the enhanced migration observed in MV4-11R cells (Fig. 3D, Supplementary Fig. S3B).

### Synergy of PF-431396 and midostaurin in TKI-resistant cells

**Fig. 4:**
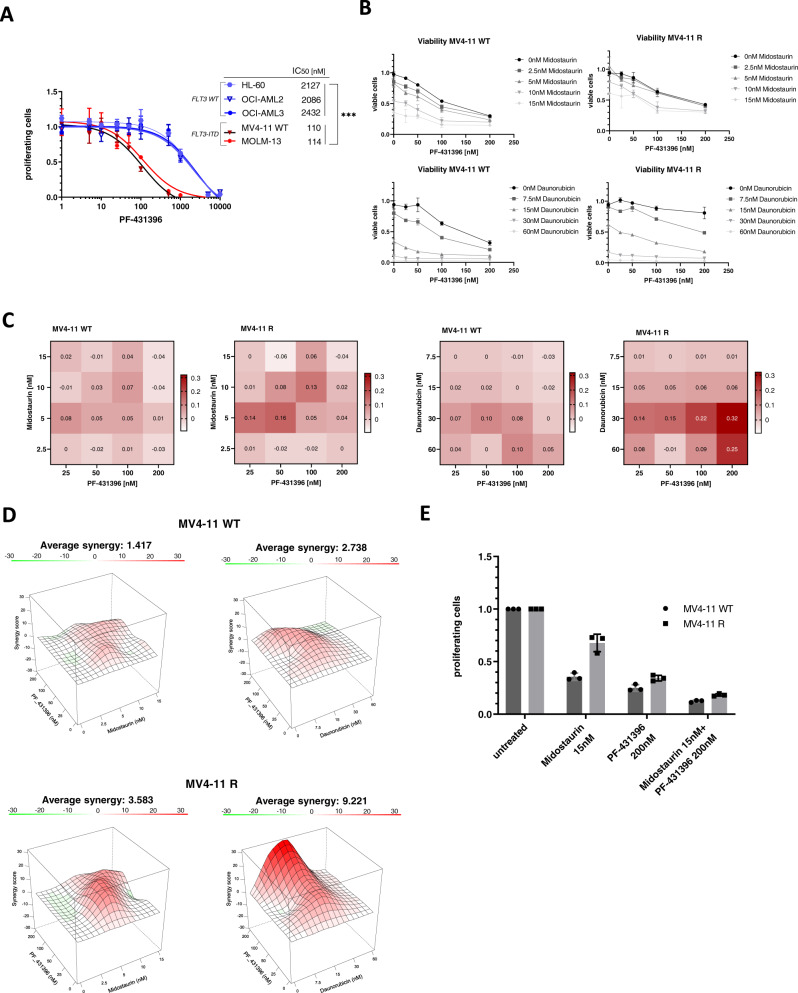
PF-431396 and midostaurin are synergistic in FLT3-mutated cells, particularly in midostaurin-resistant cells. **A** IC_50_s for PF-431396 were determined by MTS assays in different FLT3-mutated (red: MV4-11, MOLM-13) and FLT3-wildtype (blue: HL-60, OCI-AML2, OCI-AML3) cells. Depicted are means from technical triplicates ± SD. Statistical significance was assessed using unpaired two-tailed students *t*-test. ***p = 0.0007. **B** Dose-response assays for MV4-11 WT and MV4-11R cells treated with midostaurin or daunorubicin combined with PF-431396 for 72 h. Viability was assessed by staining with MTS reagent. Depicted are means from technical triplicates ± SD. **C** Dose response matrix depicting Bliss scores for the midostaurin/PF-431396 and daunorubicin/PF-431396 combination. Bliss <0 indicates antagonism, Bliss = 0 indicates the two drugs act independent; Bliss >0 indicates synergy. Bliss scores were calculated from dose response assays from Fig. 4B. **D** Synergy between midostaurin and PF-431396, as well as daunorubicin and PF-431396 in MV4-11 WT and MV4-11R cells. Depicted are Bliss average synergy scores from dose-response assays performed with the same drug concentrations as in 4B and C. **E** Dose-response assays for MV4-11 WT and MV4-11R cells treated with midostaurin combined with PF-431396 for 72 h. Viability was assessed by staining with MTS reagent. Depicted are means from technical triplicates ± SD.

We determined the IC_50_ of PF-431396 in AML cell lines. Cell lines with FLT3-ITD mutation were sensitive to the inhibitor, whereas FLT3 wildtype cell lines did not respond (Fig. 4A). Accordingly, the FLT3-ITD mutational status is of particular importance for PTK2B inhibitor sensitivity.

Both, cell migration and adhesion, were negatively affected upon addition of PF-431396. We thus tested whether the combination of midostaurin with PTK2B inhibition would enhance antiproliferative effects. The combination of the two TKIs at various concentrations were synergistic in MV4-11 cells (Fig. 4B–D). Of note, this synergy was also observed in FLT3-mutated cells for the combination of PF-431396 with daunorubicin (Fig. 4D). For FLT3-wildtype HL-60 cells, on the other hand, addition of PF-431396 did not improve response to daunorubicin (Supplementary Fig. S4A).

Synergistic effects of midostaurin and PF-431396 were more pronounced in resistant MV4-11 cells compared to parental cells. Addition of the PTK2B inhibitor largely reverted midostaurin-resistance (Fig. 4D, E).

We confirmed these effects in a MOLM-13 midostaurin-resistant cell line (MOLM-13R), also generated by repeated exposure to midostaurin. In line, we observed upregulation of LPXN upon acquired resistance (Supplementary Fig. S4B). Also in MOLM-13R we observed synergy of PF-431396 with midostaurin, daunorubicin, as well as gilteritinib (Supplementary Fig. S4C–E).

### The PTK2B inhibitor defactinib synergizes with gilteritinib in FLT3-ITD mutated cells

**Fig. 5:**
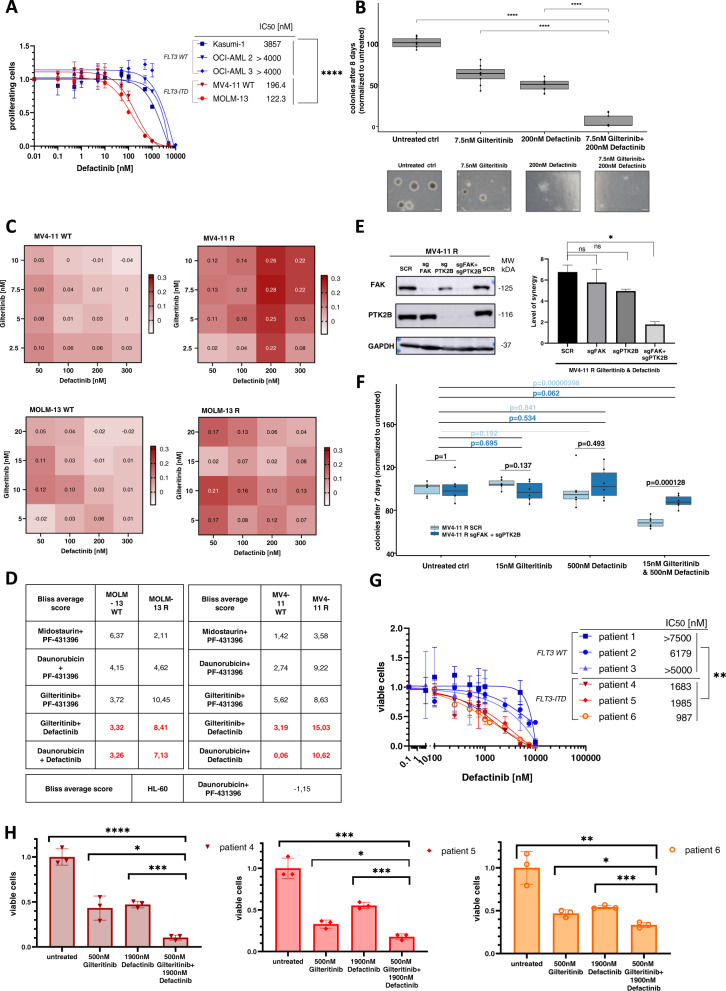
Defactinib and gilteritinib act synergistically in FLT3-ITD mutated cell lines. **A** IC_50_s for defactinib were determined by MTS assays in FLT3-mutated (red: MV4-11, MOLM-13) and FLT3-wildtype (blue: Kasumi-1, OCI-AML2, OCI-AML3) cells. Depicted are means from technical triplicates ± SD. Statistical significance was assessed using unpaired two-tailed students *t*-test. ****p = <0.0001. **B** Colony forming unit assay of MV4-11 WT cells treated with 7.5 nM gilteritinib, 200 nM defactinib and the combination. Colonies were counted after eight days of culturing. Depicted are data from three biological replicates. Scale bars represent a length of 0.4 mm. Statistical significance was assessed using unpaired two-tailed students *t*-test. ****p < 0.0001. **C** Dose response matrix depicting Bliss scores for the gilteritinib/defactinib combination. Bliss scores were calculated from dose response assays shown in Figure S5A. **D** Bliss average synergy scores of MV4-11 WT and R, MOLM-13 WT and R, and HL-60 WT with different drug combinations. **E** Left: Confirmation of single and simultaneous knockout of FAK and PTK2B in MV4-11R cells by western blot. Right: Synergy assays of MV4-11R control, as well as single knockout (FAK and PTK2B) or simultaneous knockout (FAK + PTK2B). Cells were treated with gilteritinib and defactinib for 72 h. Depicted are Bliss average synergy scores calculated from dose response assays from three technical replicates of two biological replicates. Statistical significance was assessed using unpaired two-tailed students *t*-test. *p = 0.0102 ns = not significant. **F** Colony forming unit assay of MV4-11R control and simultaneous FAK and PTK2B knockout cells treated with 15 nM gilteritinib, 500 nM defactinib or the combination of both. Depicted are triplicates of two biological replicates (third biological replicate is shown in Supplementary Fig. S5C). Statistical significance was assessed using unpaired two-tailed students *t*-test. **G** Three FLT3-ITD mutated patient samples (red) and three FLT3-WT patient samples (blue) were treated with defactinib for 72 h and viability was measured by cell counting. Depicted are three technical replicates. Statistical significance was assessed using unpaired two-tailed students *t*-test. **p = 0.0039. H. Dose-response assays for FLT3-ITD mutated patient samples #4, #5, and #6 exposed to gilteritinib and defactinib for 72 h. Viability was assessed by cell counting and normalized to untreated cells. Depicted are means from technical triplicates ± SD. Statistical significance was assessed using unpaired two-tailed students *t*-test. Patient 4: ****p < 0.0001, *p = 0.0141, ***p = 0.0002; patient 5: ***p = 0.0004, *p = 0.0119, ***p = 0.0002; patient 6: **p = 0.0039, *p = 0.0116, ***p = 0.0007.

We analyzed a second PTK2B/FAK inhibitor, i.e. defactinib, for its synergistic effect with midostaurin and other commonly used agents in AML therapy. Defactinib is currently being assessed in phase II trials for non-hematologic cancers, e.g. in the treatment of ovarian cancer and non-small cell lung cancer (NSCLC; NCT04625270, NCT04620330) [22]. In chronic lymphocytic leukemia (CLL) defactinib was shown to synergistically reduce migration capacity of CLL cells together with ibrutinib [23]. IC_50_s of defactinib in FLT3-ITD mutated cells were much lower than in FLT3 wildtype cell lines, which is in line to what was observed for PF-431396 (Fig. 5A). Gilteritinib is a TKI that is commonly used in refractory or relapsed FLT3-ITD patients, which we therefore also tested for a combinatory benefit with PTK2B/FAK inhibitors. The colony forming capacity was strongly reduced upon gilteritinib and defactinib combination treatment, as compared to treatment with only either of the drugs alone (Fig. 5B). The combination of both drugs showed synergistic effects in the FLT3-ITD mutated cell lines (Fig. 5C, Supplementary Fig. S5A, B). Effects were most pronounced in resistant MV4-11 and resistant MOLM-13 cells. Bliss average scores for this combination were higher than for midostaurin and PF-431396. Defactinib also showed synergistic effects in combination therapy with daunorubicin, again with particularly high Bliss scores in resistant cells (Fig. 5D, Supplementary Fig. S5B). Given that defactinib is a dual inhibitor with equal IC_50_s for PTK2B and FAK, we investigated the inhibition of which kinase is primarily responsible for the observed effects in FLT3-ITD mutated AML cells. PTK2B and FAK were depleted alone or together in MV4-11R cells using the CRISPR/Cas9 system (Fig. 5E). Synergy assays with gilteritinib and defactinib revealed that only the simultaneous knockout of FAK and PTK2B led to a significant reduction in synergy compared to control cells (Fig. 5E). The same was observed in colony formation assays of control and PTK2B/FAK knockout MV4-11R cells (Fig. 5F, Supplementary Fig. S5C). Single or dual knockout itself, however, did not significantly affect colony formation capacity or the response to defactinib single-agent treatment (Supplementary Fig. S5D). Our data indicate that inhibition of both, FAK and PTK2B, are required for the synergistic effect of defactinib with other TKIs or anthracyclines in FLT3-ITD AML.

We also tested the efficiency of defactinib in six primary AML patient samples (three FLT3 wildtype and three FLT3-ITD patients). Consistently, FLT3-ITD AML patient samples showed significantly lower IC_50_ values for defactinib (Fig. 5G, Supplementary Fig. S5E). Also, for FLT3-mutated patient samples gilteritinib and defactinib combination treatment was superior to treatment with either drug alone (Fig. 5H).

### PTK2B/FAK inhibitors and TKIs synergize in AML-niche models and in-vivo

**Fig. 6:**
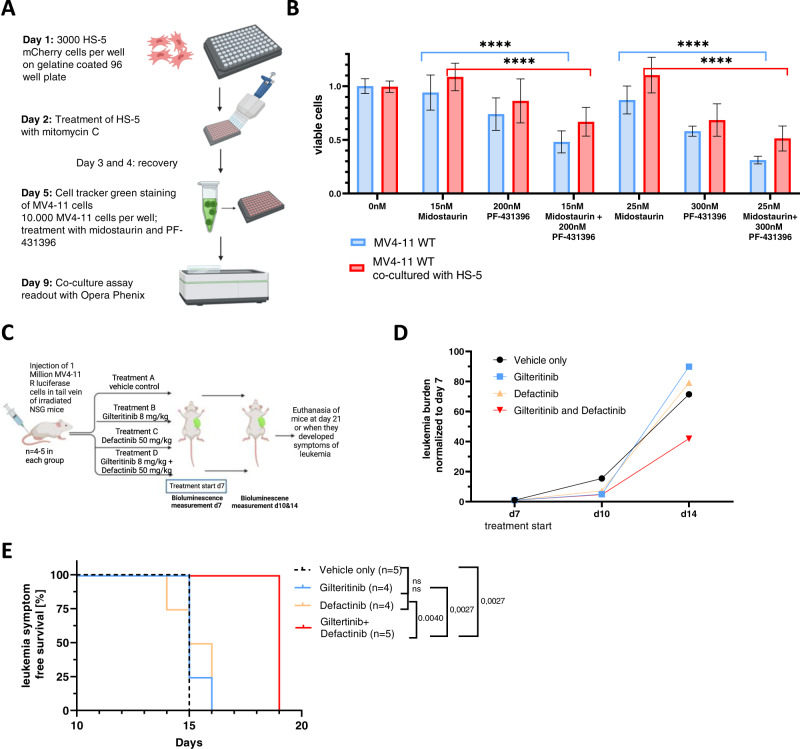
PTK2B/FAK inhibitors and TKIs synergize in AML-niche models and in-vivo. **A** Workflow scheme co-culture assay with MV4-11 WT and HS-5 stroma cells. **B** Dose response assays for MV4-11 WT cells cultured alone or together with HS-5 and treated with 15/25 nM midostaurin or 200/300 nM PF-431396 or the combination. Depicted are technical triplicates of four independent biological replicates for the first combination and technical duplicates of three biological replicates for the second combination. Statistical significance was assessed using unpaired two-tailed students *t*-test. ****p < 0.0001. **C** Workflow scheme of MV4-11R-derived xenograft experiments with gilteritinib/defactinib combination treatment or treatment with either drug alone. Vehicle only treated mice serving as control. **D** Leukemia burden and change over time in the different treatment groups of mice calculated from bioluminescence measurement. **E** Kaplan-Meier plot for time to leukemia symptom related endpoint. *p*-values were calculated by log-rank test.

Based on our data we linked emerging midostaurin-resistance, as well as the novel synergistic drug combination of FLT3- and PTK2B/FAK-inhibition to altered niche-interactions. Therefore, we assessed the synergy between midostaurin and PF-431396 in an AML-niche model. For this we performed co-culture viability assays with MV4-11 cells in the presence or absence of mesenchymal stroma cells (MSC, HS-5), exposed to combination treatment with midostaurin/PF-431396 or either drug alone (Fig. 6A, Supplementary Fig. S6A). Consistent with many prior studies, co-culturing with HS-5 cells resulted in reduced drug sensitivity of MV4-11 cells for both midostaurin and PF-431396 [24]. Also, upon co-culturing of MV4-11 cells with HS-5 cells we observed a synergistic effect of midostaurin and PF-431396. This synergy was even more pronounced than in MV4-11 cells without MSC co-cultivation (Fig. 6B, Supplementary Fig. 6B).

Finally, we assessed the benefit of addition of a PTK2B/FAK inhibitor in-vivo. We transplanted MV4-11R cells into irradiated NSG mice. Of note, the resulting leukemia was lethal to the untreated mice already after 14 days, underlining the aggressiveness of this cell line xenograft. After 7 days, bioluminescence measurement indicated engraftment and we initiated treatment with vehicle only, gilteritinib (8 mg/kg), defactinib (50 mg/kg) or gilteritinib/defactinib once per day by oral gavage (Fig. 6C). Bioluminescence imaging on day 10 and 14 revealed a lower leukemia burden in drug combination-treated mice (Fig. 6D, Supplementary Fig. S6D). Either gilteritinib or defactinib treatment alone did not impact on the aggressive course of disease. The gilteritinib/defactinib combination treated mice, however, showed a significantly longer time to leukemia symptom-related endpoint (Fig. 6E).

## Discussion

In this study, we demonstrated that induction of the PTK2B-LPXN cascade occurs early during acquired drug-resistance in FLT3-mutated AML. Inhibition of PTK2B by PF-431396 reverted a substantial number of resistance-associated alterations in the translatome, together with abrogation of resistance-associated phenotypes, such as enhanced cell migration. We revealed a synergistic effect of PTK2B/FAK inhibition with commonly used therapeutics in FLT3-mutated AML cells and found this synergy to be particularly pronounced in cells with induced TKI-resistance.

LPXN and PTK2B have previously just marginally been described in the context of AML. Depletion of LPXN in an acute monocytic leukemia cell line (SHI-1) was associated with decreased malignant proliferation and transmembrane invasion [25]. For PTK2B, a study showed that high expression levels are associated with a better prognosis in intermediate-risk AML patients [26]. For AML in general, on the other hand, TCGA data points towards an inverse correlation between PTK2B expression and overall survival (Fig. 1G). It has been shown that PTK2B interacts with both wildtype and ITD-mutated FLT3 and that FLT3-ITD inhibition induces dephosphorylation and thus inhibition of PTK2B [27]. This led to the hypothesis that FLT3-ITD inhibition might result in leukemia cell detachment from the bone marrow niche through PTK2B inhibition. In line, our findings closely link PTK2B to ITD-mutated FLT3 in the context of drug resistance.

Upregulation of LPXN and PTK2B in our generated midostaurin-resistant cells was associated with significantly affected cell migration and adhesion. Further, both LPXN and PTK2B are upregulated in LSCs of FLT3-ITD mutated patients compared to non-LSCs [19]. These findings point towards a novel PTK2B-LPXN axis in CAM-DR by altered niche interactions in the context of LSC properties in FLT3-ITD AML. Consistently, we observed an even more pronounced synergy of FLT3- and PTK2B/FAK-inhibition in AML cells upon co-cultivation with mesenchymal stroma cells. Despite the uncovered close link between PTK2B/FAK inhibition and FLT3-ITD mutation, there was no apparent correlation of either LPXN or PTK2B expression with the FLT3 mutational status in our proteome, TMA or published TCGA data. Vice versa, high PTK2B or LPXN expression alone were not sufficient to predict sensitivity to PTK2B/FAK inhibition. This suggests that both FLT3 mutation and PTK2B/LPXN upregulation are important for TKI drug resistance and for PTK2B/FAK inhibitor sensitivity.

Our data proposes that a PTK2B/FAK inhibitor may constitute a beneficial additive therapeutic compound in both, treatment-naïve and relapsed/refractory FLT3-mutated AML. Pre-clinical in-vivo experiments of PF-431396 found excellent tolerability, especially with no overt bone marrow microenvironment toxicity [28]. Defactinib, on the other hand, is already being evaluated in several phase II trials for non-hematologic cancers (NCT04625270, NCT04620330), where it has been shown to be well tolerated [22, 29]. Consistently, we could show in this study that gilteritinib and defactinib prolonged time to leukemia symptom-related endpoint in-vivo, even in the case of a highly resistant cell line with a particularly aggressive course of disease. Defactinib has recently been granted breakthrough therapy designation by the FDA for the treatment of ovarian cancer, which thus could facilitate a timely investigation of the benefits of PTK2B/FAK inhibition also in FLT3-ITD mutated AML patients, based on the results of this study.

Taken together, our findings indicate that PTK2B/FAK inhibitors could target important FLT3 mutation-associated niche interactions in AML, thus representing novel putative combination partners for FLT3 inhibitors.

## Supplementary information

Supplementary Material

Supplementary Table S1

## Data Availability

Limma analyses for total and nascent proteomics experiments are included in this published article, further datasets are available from the corresponding author on reasonable request.
