# Supplementary material for: Protein tyrosine kinase 2b inhibition reverts niche-associated resistance to tyrosine kinase inhibitors in AML
Source: Leukemia. 2022 Sep 2;36(10):2418–29. doi: 10.1038/s41375-022-01687-x (PMC9522596; doi:10.1038/s41375-022-01687-x)
Supplement: Supplementary file 1 — Supplementary Material [file 41375_2022_1687_MOESM1_ESM.pdf]

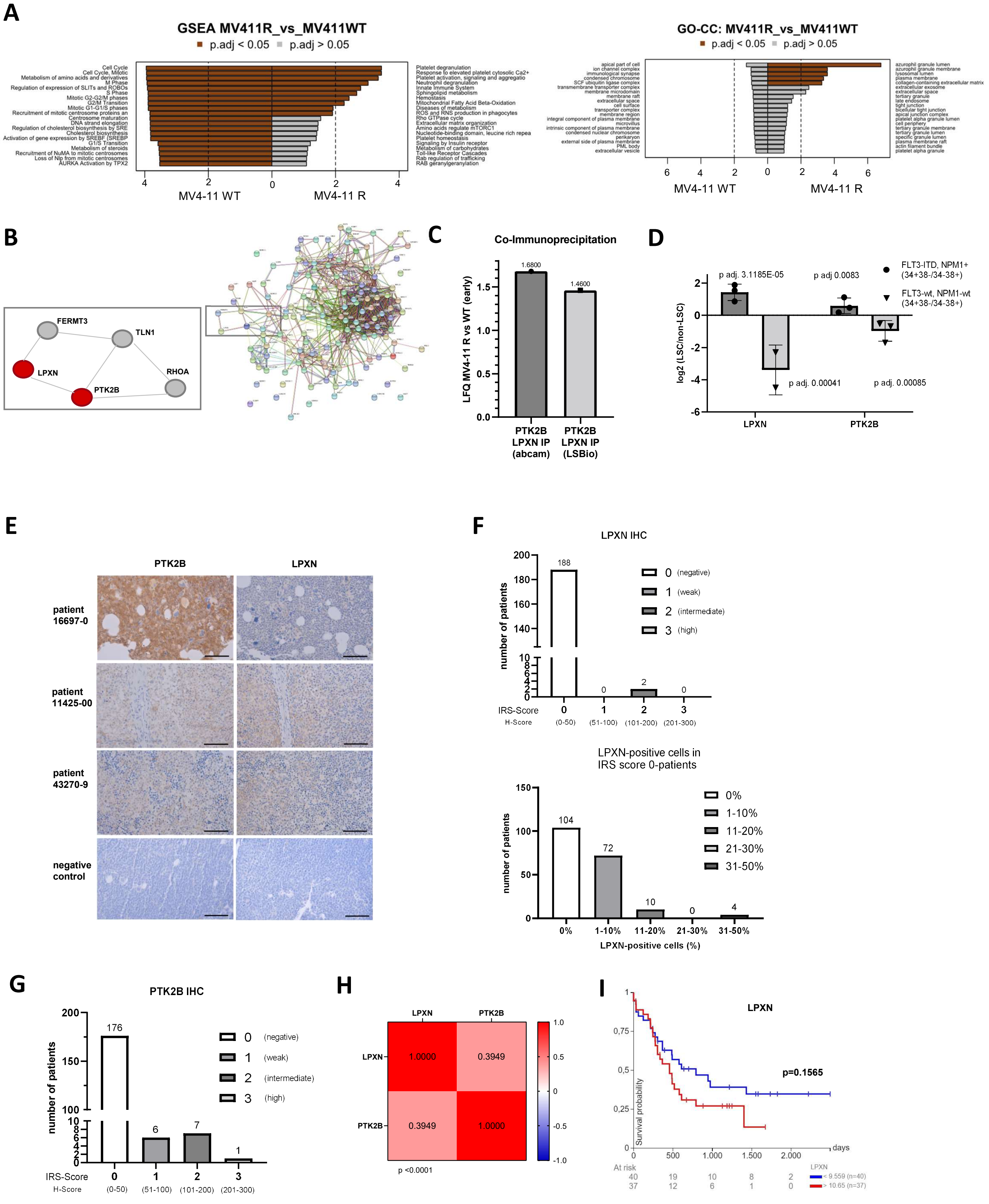

Supplementary Figure S1.

Supplementary Figure 1, related to Figure 1. LPXN is induced during acquired midostaurin-resistance and is phosphorylated by PTK2B

A. Total proteome analysis by mass spectrometry of MV4-11 parental and MV4-11 R (late). Left: gene set enrichment analysis (GSEA), MV4-11 R versus parental. Right: Gene ontology (GO) analysis for cellular component (CC).

B. Co-immunoprecipitation of endogenous LPXN in MV4-11 cells. String (https://string-db.org/) network analysis of proteins interacting with LPXN and PTK2B in MV4-11 WT cells

C. Interaction of LPXN and PTK2B in MV4-11 R (early) vs MV4-11 parental cells estimated by LFQs in Co-IP-MS experiments using two different anti-LPXN antibodies (Abcam and LSBio).

D. LPXN and PTK2B protein expression in LSCs and non-LSCs of FLT3-WT and FLT3-ITD patients. Data are derived from Raffel et. al. 2017.

E. Representative immunohistochemistry staining of bone marrow from AML patients at diagnosis with LPXN and PTKB antibodies. LPXN was detected in 88 bone marrow specimens from patients at time point of diagnosis. For 14 patient biopsies IRS for PTK2B positive cells was > 1. Primarily all-or-nothing pattern of expression with either absence (H-score=0) or strong expression was observed (H-score>150).

F. Upper: Immunoreactive Score by Remmele (IRS) for leupaxin immunohistochemical staining of 190 patient bone marrow samples. Lower: Number of patients with LPXN positive cells within the IRS score 0 group.

G. Immunoreactive Score by Remmele (IRS) for PTK2B immunohistochemical staining of 190 patient bone marrow samples.

H. Pearson correlation coefficient of LPXN and PTK2B expression in TMA samples. r=0.3949, p< 0.0001.

I. Kaplan–Meyer plot for overall survival of patients with low and high LPXN mRNA expression (TCGA LAML data set). Statistical significance was determined by log-rank test.

Supplementary methods 1, related to Figure S1. Figure 1. LPXN is induced during aquired midostaurin-resistance and is phosphorylated by PTK2B

Tissue microarray and immunohistochemistry

A tissue microarray with 219 formalin fixed and paraffin embedded (FFPE) bone marrow biopsies of AML patients was available. IHC stainings were performed on a Bond III automated immunostainer (Leica Biosystems) using the Bond Polymer Refine Detection Kit (DS9800-CN). The primary antibodies directed against Leupaxin (1:500, ab181621, Abcam) and PYK2 (1:250, ab81266, Abcam)) were applied as recommended by the manufacturer. Immunostaining was assessed by two investigators blinded to additional pathological and clinical data using Zeiss Axioscope 5 microscope (Carl Zeiss Microscopy GmbH). Semi-quantitative evaluation was carried out using the Histo-scoring system (Detre 1995 PMID: 7490328). TMA of 190 patients could be analyzed. Written informed consent was obtained from all patients. The study was approved by the local Institutional Review Board of the University of Münster.

Supplementary methods 1, related to Figure 1. LPXN is induced during aquired midostaurin-resistance and is phosphorylated by PTK2B

Steady-state proteome analyses

Cells were lysed in 50mM ammonium bicarbonate containing 0.1% Rapigest (Waters) and sonicated samples were reduced with dithiothreitol, alkylated with chloroacetamide and digested with trypsin and rLysC (Promega). After acidification samples were spun and supernatants were used for injections. A peptide amount corresponding to 1µg was analyzed on a Tri-Hybrid Orbitrap Fusion mass spectrometer (Thermo Fisher Scientific) operated in data-dependent acquisition mode with HCD fragmentation. RAW data were processed with MaxQuant (1.6.2.6) (1) using default settings. Only proteins with at least 1 unique peptide were considered as identified and normalized LFQ values were used for quantitative comparative analyses. The gene set enrichment analyses (GSEA) were performed using R package fgsea (2) (version 1.6.0) with a p-value ranking of proteins, gene sets defined by the REACTOME pathway database (R package ReactomePA version 1.24.0) (3)

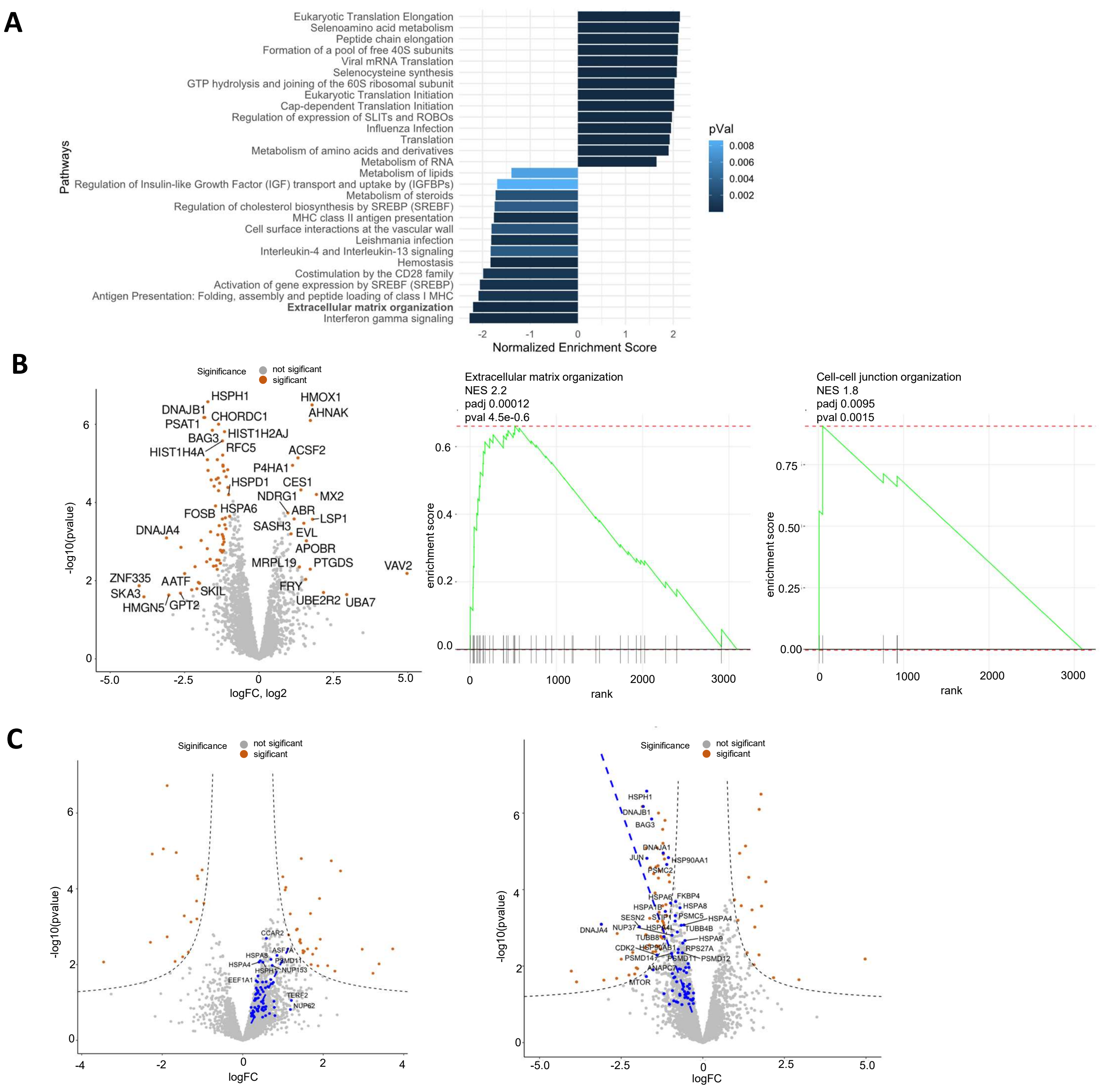

Supplementary Figure S2.

Supplementary Figure 2, related to Figure 2. Alterations in protein homeostasis upon midostaurin-resistance and PTK2B inhibition

A. Nascent proteomics of MV4-11 parental and resistant cells. GSEA of changes in translation between resistant and parental cells.

B. Nascent proteomics of MV4-11 R cells with or without PF-431396 treatment (16h, 300nM). Left: volcano plot of differentially translated proteins, significantly altered proteins are labelled in orange (log2FC>1, p-value<0.05). Right: GSEA plots for gene set "extracellular matrix organization" and "cell-cell junction organization" in MV4-11 R treated with 300nM PF-431396 compared to R cells.

C. Nascent proteomics in MV4-11 R vs MV4-11 WT (left) and MV4-11 R +inhibitor vs MV4-11 R (right). Proteins involved in cellular response to external stimuli are highlighted in blue.

Supplementary methods 2, related to Figure 2. Alterations in protein homeostasis upon midostaurin-resistance and PTK2B inhibition

Protein degradation/stability analysis by SILAC and LC-MS/MS

Wildtype and resistant cells were cultured in heavy (HV) or intermediate (IM) SILAC media for more than five passages. SILAC media were then changed to light medium. Samples were harvested at timepoints 0, 6, 12, 24, 36, and 48 hours after medium change. Cells were washed with PBS, resuspended in RapiGest™ SF surfactant, and sonicated. Corresponding differentially labeled wildtype and resistant cell lysates were mixed at equal protein amount ratios. To exclude labeling-dependent effects, this was performed in replicates grown in the opposite HV/IM combination. Samples underwent reduction, alkylation, and digestion, as described for total proteome analysis. LC-MS/MS was subsequently performed on an Orbitrap Fusion mass spectrometer. MaxQuant output was analyzed focusing on normalized HV/IM ratios of proteins.

Nascent proteomics

Nascent proteomics studies were performed as described before (4). Cells were pulse-labeled for 6 hours with AHA- and SILAC-amino acid containing medium. Compared conditions were labeled with different SILAC medium (HV or IM). After harvest, washing and cell lysis, equal amounts (750 µg each) from different conditions and SILAC labels ((HV)/(IM)) were combined and proceeded to AHA-enrichment. Newly synthesized proteins were enriched using the Click-iT® Protein Enrichment Kit (Invitrogen, Schwerte, Germany), applying the vendor's protocol with slight modifications as described.

A

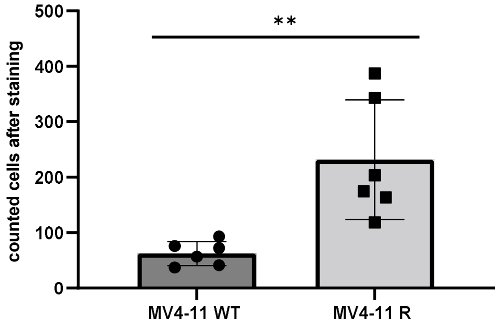

B

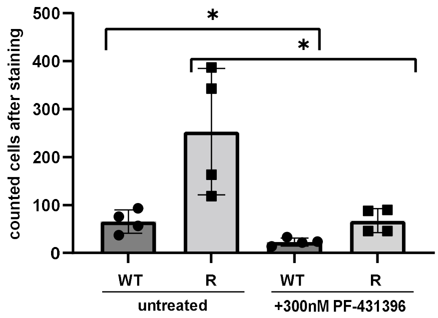

Supplementary Figure S3.

Supplementary Figure 3, related to Figure 3. PTK2B/FAK inhibitor PF-431396 abolishes enhanced cell migration in midostaurin-resistant cells

A. Cell migration assays were performed with MV4-11 parental and MV-11 R cells which migrated for 24h. Cell counting results after staining and washing procedure. Depicted are means from three individual field counts of technical duplicates of three biological replicates ± SD. Statistical significance was assessed using unpaired two-tailed students t-test. ***p*= 0.0037.

B. Enhanced migration of MV4-11 R cells is abolished after 300nM PF-431396 treatment of R cells. Cell counting results after staining and washing procedure. Depicted are means from three individual field counts from duplicates of two biological replicates ± SD. Statistical significance was assessed using unpaired two-tailed students t-test. **p*= 0.0154; **p* =0.0325.

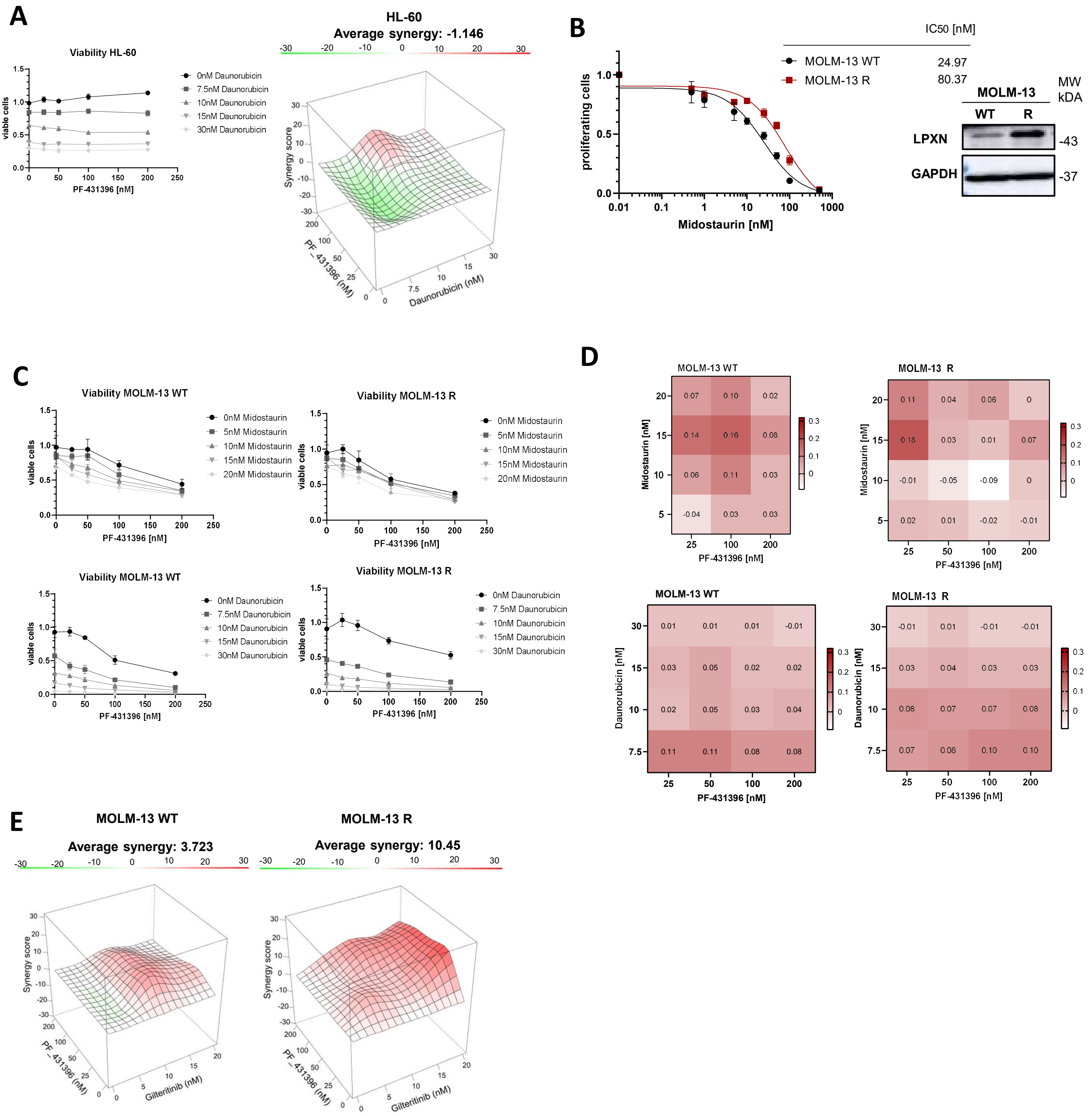

Supplementary Figure S4.

Supplementary Figure 4, related to Figure 4. PF-431396 and midostaurin are synergistic in FLT3-mutated cells, particularly in midostaurin-resistant cells

A. Left: Dose response assay for HL-60 cells treated with daunorubicin (0, 7.5, 10, 15, 30nM) and PF-431396 (0, 25, 50, 100, 200nM) for 72h. Viability was assessed by staining with MTS reagent. Depicted are three technical replicates. Right: Bliss score between daunorubicin and PF-431396 in FLT3-WT HL-60 cells. Depicted are Bliss average scores from dose response assays.

B. Left: MOLM-13 cells were exposed to increasing concentrations of midostaurin for several weeks. Acquired midostaurin-resistance was analyzed by MTS assays. Depicted are means from technical triplicates ± SD. Right: Induction of LPXN protein in MOLM-13 R cells analyzed by western blot.

C. Dose response assays for MOLM-13 WT and MOLM-13 R cells treated with midostaurin (0, 5, 10, 15, 20nM) or daunorubicin (0, 7.5, 10, 15, 30nM) and PF-431396 (0, 25, 50, 100, 200nM) for 72 h. Viability was assessed by staining with MTS reagent. Depicted are three technical replicates.

D. Dose response matrix depicting Bliss scores for the midostaurin/PF-431396 and daunorubicin/PF-431396 combination. Bliss scores were calculated from dose response assays depicted in S4C.

E. Synergy between gilteritinib and PF-431396 is assessed in MOLM-13 R cells. Depicted are Bliss average scores from dose response assays with gilteritinib (0, 5, 10, 15, 20nM) and PF-431396 (0, 25, 50, 100, 200nM).

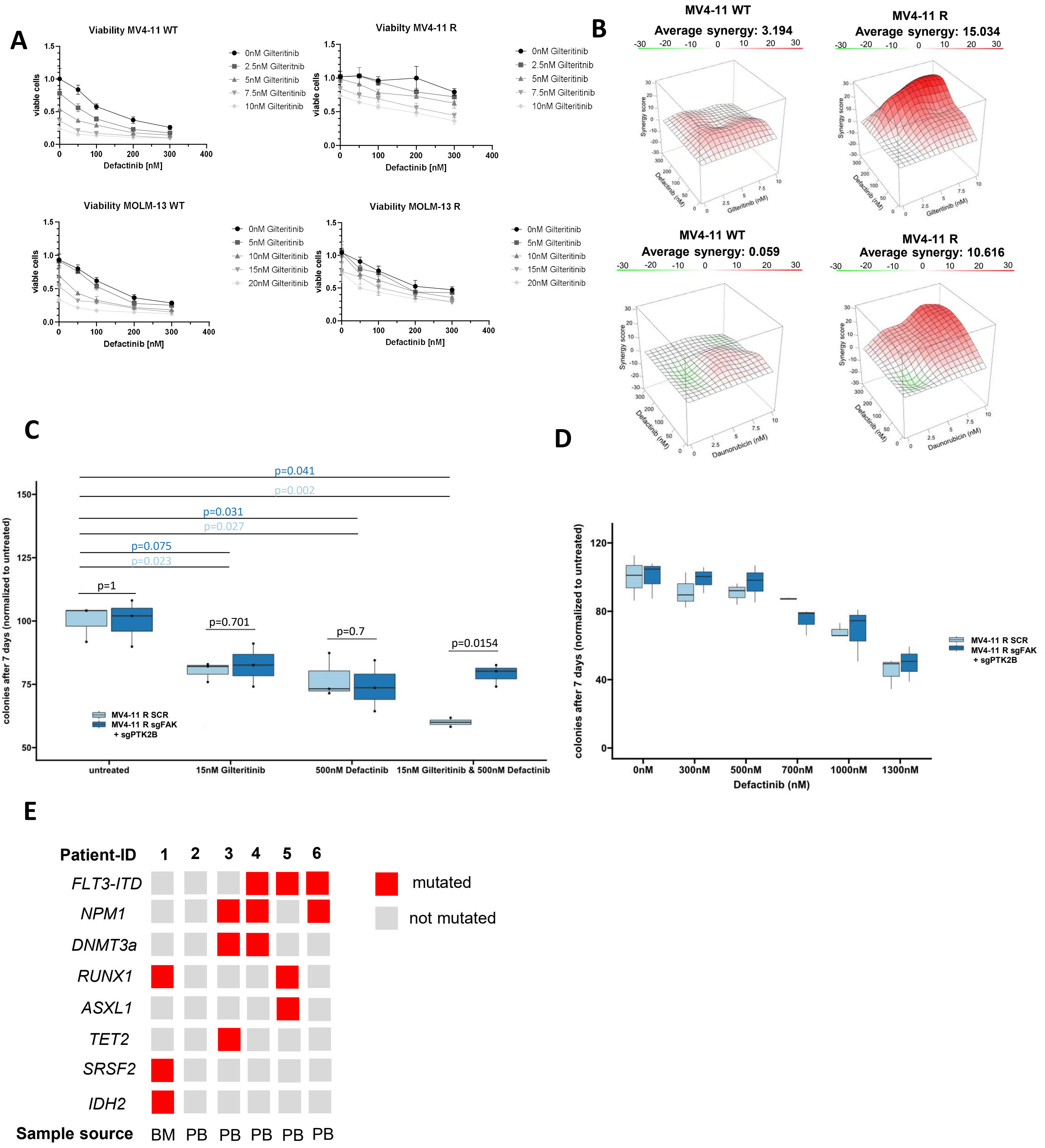

Supplementary Figure S5.

Supplementary Figure 5, related to Figure 5. Defactinib and gilteritinib act synergistically in FLT3-ITD mutated cell lines

A. Dose response assays for MV4-11 WT and MV4-11 R cells treated with gilteritinib (0, 2.5, 5, 7.5, 10nM) and defactinib (0, 50, 100, 200, 300nM) as well as MOLM-13 WT and MOLM-13R cells treated with gilteritinib (0, 5, 10, 15, 20nM) and defactinib (0, 50, 100, 200, 300nM) for 72 h. Viability was assessed by staining with MTS reagent.

B. Synergy between gilteritinib and defactinib as well as daunorubicin and defactinib in MV4-11 R and MV4-11 parental cells. Depicted are Bliss average scores derived from dose response assays given in Figure S5A.

C. Left: Colony formation assay of MV4-11 R SCR and FAK+PTK2B dual knockout cells treated with 15nM gilteritinib, 500nM defactinib or the combination. 300 cells were seeded in methylcellulose and counted after seven days of culturing. Depicted are means from three technical triplicates. Statistical significance was assessed using unpaired two-tailed students t-test.

D. Colony formation assay of MV4-11 R control vs MV4-11 R with simultaneous FAK and PTK2B knockout treated with different concentrations of defactinib (300, 500, 700, 1000, 1300nM) alone.

E. Mutational status and sample source (BM or PB) of primary samples shown in Figure 5F.

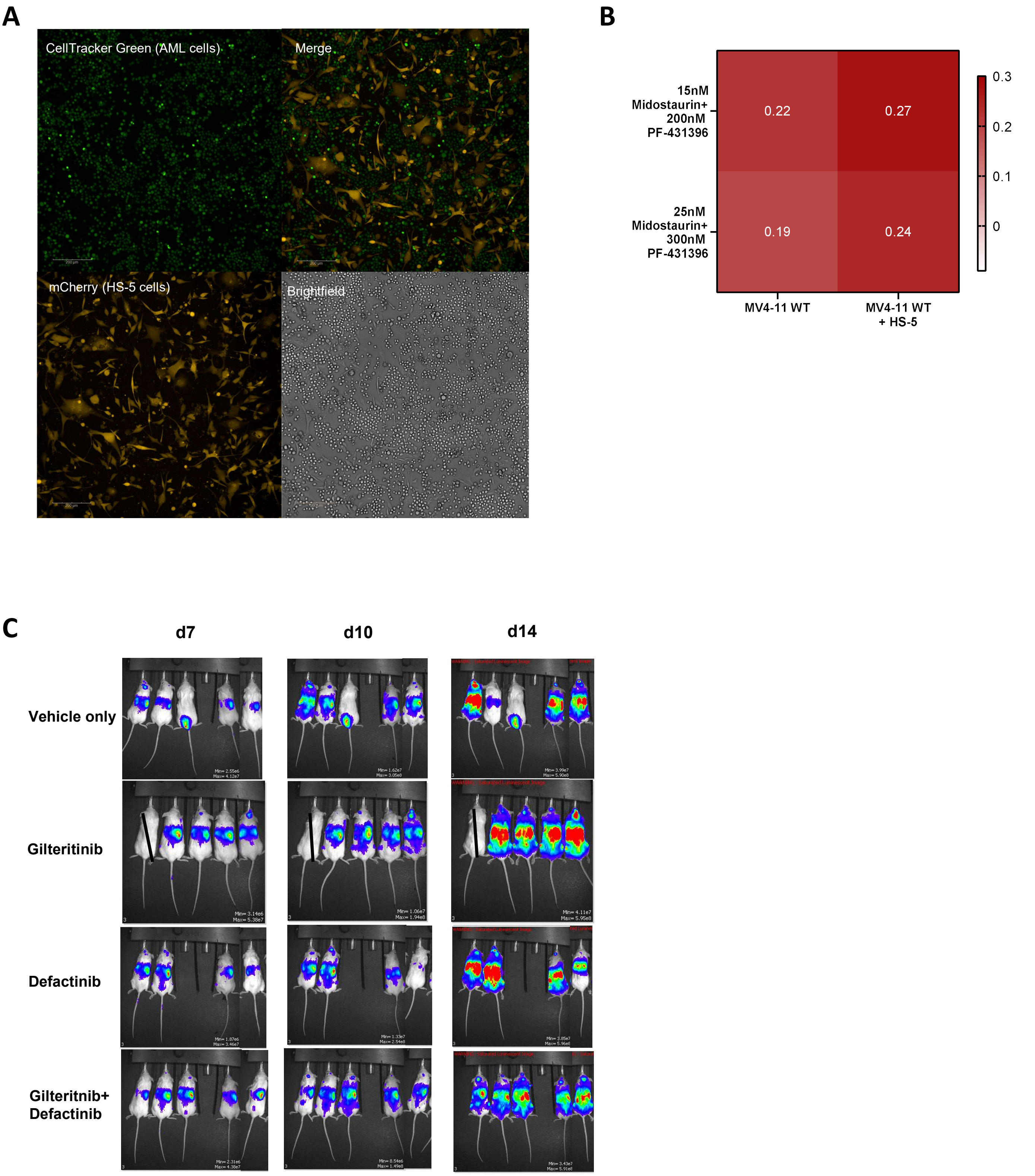

Supplementary Figure S6.

Supplementary Figure 6, related to Figure 6. PTK2B/FAK inhibitors and TKIs synergize in AML-niche models and in-vivo

A. Representative microscopy images of co-culture assays with MV4-11 WT and HS-5 cells. Pictures were taken with 10x air objective.

B. Bliss synergy scores for MV4-11 WT cells cultured alone and together with HS-5 and treated with 15nM Midostaurin, 200nM PF-431396 or the combination or 25nM Midostaurin, 300nM PF-431396 or the combination. Depicted are data of at least two technical replicates of at least three biological replicates.

C. Bioluminescence images of the treatment groups of mice at day 7 (treatment start), day 10 and day 14.

Supplementary methods 6, related to Figure 6. PTK2B/FAK inhibitors and TKIs synergize in AML-niche models and in-vivo

Co-culture assays:

3000 HS-5 mCherry cells were plated out per well in black 96well plates coated with 0.1% gelatine (0621.1, Carl Roth GmbH). After 24h HS-5 cells were treated with 1,3µg/ml mitomycin C (10286710, ThermoFisher Scientific) to suppress cell proliferation. After two days of recovery 10.000 AML cells stained with 2µM CellTracker™ Green CMFDA dye (C7025, ThermoFisher Scientific) were seeded on top. Cells were treated with 15nM, 25nM midostaurin as well as 200nM, 300nM PF-431396 and the combination. 96h later co-culture assays were analyzed with confocal microscopy (Opera Phenix). Images were taken with 10x air objective and image analysis and counting of AML cells was performed using harmony software.

References:

1. Cox J, Mann M. MaxQuant enables high peptide identification rates, individualized p.p.b.-range mass accuracies and proteome-wide protein quantification. Nat Biotechnol. 2008;26(12):1367-72.

2. Sergushichev AA. An algorithm for fast preranked gene set enrichment analysis using cumulative statistic calculation. bioRxiv. 2016:060012.

3. Yu G, He QY. ReactomePA: an R/Bioconductor package for reactome pathway analysis and visualization. Mol Biosyst. 2016;12(2):477-9.

4. Eichelbaum K, Winter M, Berriel Diaz M, Herzig S, Krijgsveld J. Selective enrichment of newly synthesized proteins for quantitative secretome analysis. Nat Biotechnol. 2012;30(10):984-90
